# Smoking‐aggravated oral candidiasis: Nrf2 pathway dampens NLRP3 inflammasome

**DOI:** 10.1111/jcmm.16901

**Published:** 2021-09-05

**Authors:** Marwan Osman, Nicolas Papon

**Affiliations:** ^1^ Laboratoire Microbiologie Santé et Environnement (LMSE) Doctoral School of Sciences and Technology Faculty of Public Health Lebanese University Tripoli Lebanon; ^2^ Department of Population Medicine and Diagnostic Sciences College of Veterinary Medicine Cornell University Ithaca New York USA; ^3^ GEIHP SFR ICAT University of Brest University of Angers Angers France

**Keywords:** Candida albicans, immunity, NLRP3 inflammasome, Nrf2, oxidative stress, smoking

## Abstract

While cigarette smoke compounds are known to have immunosuppressive effects on the oral mucosa, the relationship between in vivo immune dysfunction caused by smoking and the development of oral Candida infections remains largely unexplored. In a recent issue of The Journal of Cellular and Molecular Medicine, Ye and colleagues provide evidence that smoking increases oral mucosa susceptibility to Candida albicans infection via the activation of the Nrf2 pathway, which in turn negatively regulates the NLRP3 inflammasome. This opens new perspective in considering Nrf2 as a relevant target for smoking‐induced C. albicans‐related oral diseases.

Cigarette smoke (CS) contains a complex mixture of oxidants and numerous reactive oxygen species (ROS) predisposing humans to several mild‐to‐severe oral health problems, including oropharyngeal candidiasis (OPC) and oral leukoplakia (OLK). OPC, commonly referred to as ‘thrush’, corresponds to infections of various oral mucosal sites and is characterized by Candida yeasts overgrowth and invasion of superficial tissues while OLK encompasses white patches or plaques potentially malignant that develops in the oral cavity. Oxidative stress and imbalance of the cellular redox state induced by smoking can change the oral microbiome and damage the structure and function of the oral epithelial barrier, the first‐line defence against fungal infections.^1^ Smoking may also promote Candida albicans pathogenicity by enhancing fungal adhesion, growth and biofilm formation.^2^ However, the exact metabolic mechanism by which smoking increases susceptibility of the oral mucosa to candidiasis has not yet been explored. In addition, although CS compounds are known to have immunosuppressive effects on the innate immune response by decreasing phagocytic activity and affecting chemotaxis, kinesis, and cell signalling,^3^ there is a lack of information on the relationship between in vivo immune dysfunction caused by smoking and the development of C. albicans oral infection. In a recent issue of The Journal of Cellular and Molecular Medicine, Ye and coworkers^4^ provide new insight into a key role of the nuclear factor erythrocyte 2–related factor 2 (Nrf2) signalling pathway in smoking‐aggravated C. albicans oral infection. Importantly, this study extends their previous research,^5^ which demonstrated that CS suppresses C. albicans‐induced NOD‐like receptor family pyrin domain containing 3 (NLRP3) inflammasome activation and pro‐inflammatory cytokine production in a rat model.

A preliminary series of experiments first confirmed what was expected, reporting that smoking is able to enhance the proliferation of C. albicans in experimental and clinical models. The growth of C. albicans was accompanied by a marked oxidative stress on epithelial cells revealed not only by an accumulation of ROS and malondialdehyde but also by a decrease in reduced (GSH) and oxidized (GSSG) glutathione ratio (GSH/GSSG) in both CS extract‐treated C. albicans and immortalized human oral mucosa epithelial cells (Leuk1) co‐culture and CS‐treated rat infection models. Nrf2 is well documented to act in epithelial cells as an oxidative stress–sensing protein that controls over 100 genes by recognizing the antioxidant response element enhancer sequence. Here, Ye and colleagues found that in response to oxidative stress generated by smoking, Nrf2 is phosphorylated and translocated into the nucleus allowing the activation of the expression/activity of its target antioxidant proteins, including NAD(P)H dehydrogenase quinone‐1 (NQO‐1), haem‐oxygenase‐1 (HO‐1) and superoxide dismutase (SOD) (Figure 1). These findings were confirmed by silencing Nrf2, which led to an increase in ROS production and inhibition in the antioxidative response. In contrast to previous reports,^6^ this study showed that C. albicans infection alone is unable to alter ROS levels, GSH/GSSG ratio, Nrf2 status or antioxidant enzyme expression in both models. This discrepancy is probably due to the difference in C. albicans strains, type of infected cells and exposure periods. As reported previously in other bacterial and viral infections, Nrf2 may have harmful effects on host immunity. In this regard, in vitro experiments then emphasized that Nrf2 not only negatively regulates the expression levels of NLRP3 inflammasome components and caspase‐1 p20 but also reduces the secretion of pro‐inflammatory cytokines (IL‐1β, IL‐6, TNF‐α and IL‐18), which in turn attenuate oral mucosa antifungal immunity and promote oropharyngeal candidiasis and OLK (Figure 1). Interestingly, the pathophysiological relevance is provided by the observation that smokers belonging to both healthy individuals and patients with clinically suspected OLK groups showed a similar health outcome, displaying an increase in Nrf2 levels associated with a decrease in NLRP3, IL‐1β and IL‐18 in their oral mucosal tissues. Albeit the availability of some studies describing the role of ROS and NQO‐1 to regulate the interaction between Nrf2 and NLRP3, the specific molecular mechanism of this antagonistic relationship remains unclear and obviously deserves to be accurately investigated.

**FIGURE 1:**
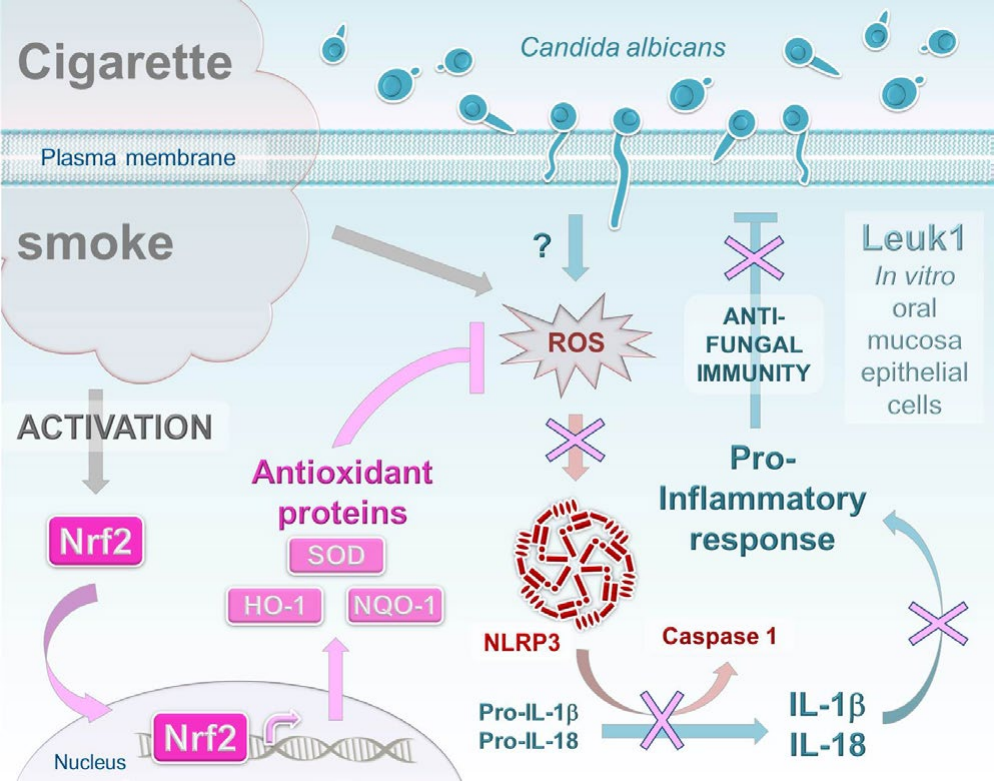
Smoking increases oral mucosa susceptibility to Candida albicans infection via the activation of the Nrf2 pathway, which in turn negatively regulates the NLRP3 inflammasome: a proposed model in oral epithelial cells. In response to oxidative stress generated by smoking, Nrf2 is phosphorylated and translocated into the nucleus allowing the activation of the expression/activity of its target antioxidant proteins, including NAD(P)H dehydrogenase quinone‐1 (NQO‐1), haem‐oxygenase‐1 (HO‐1) and superoxide dismutase (SOD). Importantly, although some studies have described the role of ROS and NQO‐1 in regulating the interaction between Nrf2 and NLRP3, the specific molecular mechanism of this antagonistic relationship remains unclear

In sum, by using three established smoking and C. albicans interaction in vivo and in vitro models, Ye and colleagues show a link between the smoking‐aggravated oral mucosa susceptibility to C. albicans infection and the activation of the Nrf2 pathway, which in turn negatively regulates the NLRP3 inflammasome. Accumulating experimental and clinical evidences showed that the Nrf2 pathway is involved in a wide range of infectious and non‐communicable diseases.7 To potentially prevent diseases, most cases have suggested pharmacological activation of Nrf2, with the exception of cancer which, in theory, requires inhibition of Nrf2 instead.8 In such a perspective, and based on the herein reported findings, the inhibition of the Nrf2 pathway could be thus considered as a potential therapeutic target to prevent oropharyngeal candidiasis among smokers. This enlightening article is in line with the current trend pinpointing the modulation of the Nrf2 and NLRP3 inflammasome activity as a promising strategy for the management of infectious diseases.^7^, ^8^, ^9^ Down‐regulation of Nrf2 coupled with up‐regulation of NLRP3 inflammasome and the subsequent pro‐inflammation appear essential to reduce the pathogenesis of C. albicans and prevent smoking‐induced infection progression. Nevertheless, the suppression of Nrf2 and its associated inflammation is a double‐edged sword for hosts to defence against fungal infection. Therefore, further studies are also needed to determine the boundary between the beneficial and potentially harmful effects of Nrf2 and NLRP3 activation. Finally, since the frequency of non‐albicans Candida species is globally increasing,^10^ similar investigations targeting other Candida species, particularly Candida dubliniensis, Candida tropicalis and Candida glabrata, are recommended. In this respect, a recent transcriptome study revealed distinct Candida species‐specific pathogenicity and transcriptional patterns influencing the host responses during infection of human epithelial cells.^11^

In conclusion, while smoking is well known to compromise the immune system of the oral mucosa, few studies focused on the in vivo immune dysfunction caused by this bad habit. Nrf2 was suggested as a double‐face protein which, on the one hand, protects the structure and function of epithelial cells against ROS, and on the other hand, attenuates oral epithelial antifungal immunity and promotes oral mucosal susceptibility to C. albicans infections. This study definitively opens the door for future investigations, not only to determine the exact mechanism by which the Nrf2 pathway regulates the NLRP3 inflammasome in this specific pathophysiological context but also to figure out Nrf2 as a key regulator and potential therapeutic target for smoking and Candida‐related oral diseases.

## CONFLICT OF INTEREST

The authors confirm that there are no conflicts of interest.

## AUTHOR CONTRIBUTIONS

**Marwan Osman:** Writing‐original draft (lead). **Nicolas Papon:** Writing‐review & editing (lead).

## Data Availability

Data sharing not applicable to this article as no datasets were generated or analysed during the current study
